# Hematopoietic Origin of Murine Lung Fibroblasts

**DOI:** 10.1155/2015/159713

**Published:** 2015-06-21

**Authors:** Lindsay T. McDonald, Meenal Mehrotra, Amanda C. LaRue

**Affiliations:** ^1^Research Services, Ralph H. Johnson Department of Veterans Affairs Medical Center, Charleston, SC 29401, USA; ^2^Department of Pathology and Laboratory Medicine, Medical University of South Carolina, Charleston, SC 29425, USA; ^3^Hollings Cancer Center, Medical University of South Carolina, Charleston, SC 29425, USA

## Abstract

Multiple origins, including the bone marrow, have been suggested to contribute to fibroblast populations in the lung. Using bone marrow reconstitution strategies, the present study tested the hypothesis that the bone marrow hematopoietic stem cell (HSC) gives rise to lung tissue fibroblasts *in vivo*. Data demonstrate that the nonadherent bone marrow fraction is enriched for CD45^+^ HSC-derived cells and was able to reconstitute hematopoiesis in lethally irradiated animals. Analysis of peripheral blood and lung tissues from engrafted mice demonstrated the ability of this population to give rise to CD45^+^/Discoidin-Domain Receptor-2^+^ (DDR2) circulating fibroblast precursors (CFPs) in blood and fibroblast populations in lung. An HSC origin for lung fibroblasts was confirmed using a novel clonal cell transplantation method in which the bone marrow is reconstituted by a clonal population derived from a single HSC. Together, these findings provide evidence for an HSC contribution to lung fibroblasts and demonstrate a circulating intermediate through the CD45^+^/DDR2^+^ HSC-derived CFP.

## 1. Introduction

Several studies have suggested a bone marrow origin for a subpopulation of lung fibroblasts [1, 2]; however the stem cell in the bone marrow that gives rise to this population remains unclear. Fibrocytes, circulating cells with the potential to differentiate into mature fibroblasts [3], have been identified in many tissues including lung [4]. These cells have been shown to express hematopoietic markers including CD45 and CD34, suggesting an origin from the hematopoietic stem cell (HSC); however, their mesenchymal nature has brought into light the possibility that the origin of this population may be through the mesenchymal stem cell (MSC). Examination of lung fibroblasts in pulmonary disease such as pulmonary fibrosis has led some to question whether proliferation of the resident lung population alone could explain the relative prominence and heterogeneity of fibroblasts found in the fibrotic foci that characterize this disease (reviewed in [5]). In support of an extrapulmonary origin of lung fibroblasts and myofibroblasts, studies have shown multiple sources of fibroblasts including an MSC origin [6], an origin through epithelial to mesenchymal transformation (EMT) (reviewed in [7]), endothelial to mesenchymal transformation (endoMT) [8], or through resident fibroblast contribution ([9] and reviewed in [10]). However, to our knowledge, to date, there have been no direct lineage tracing studies addressing the origin of lung fibroblast populations. These studies have resulted in several key unanswered questions in the study of lung pathophysiology including the following: What is the origin of lung fibroblasts? What is the bone marrow progenitor population that gives rise to these cells? And what is the phenotype and functional role of the recruited cells based on origin in both normal and disease states? (reviewed in [5]). Investigating these questions has the potential to uncover hitherto underappreciated differences and similarities between pulmonary fibroblasts of multiple origins as well as to inform research directed at elucidating the differentiation and maturation pathways of these populations towards the ultimate goal of impacting human health and disease.

Recent studies by our laboratory (reviewed in [11, 12]) and others (reviewed in [13]) have demonstrated greater plasticity of bone marrow HSCs than previously appreciated. Through direct lineage tracing studies, using a unique clonal cell transplantation method in which a clonal population derived from a single EGFP^+^ HSC is transplanted into lethally irradiated mice, our laboratory has demonstrated an HSC origin for a number of mesenchymal cell types including adipocytes, chondrocytes, osteoblasts/osteocytes, and fibroblasts (reviewed in [11]). In particular, our laboratory has previously demonstrated that HSCs give rise, through a nonfusion mechanism [14–18], to bone marrow colony forming unit fibroblasts (CFU-F) [19], circulating fibrocytes/fibroblast precursors [19, 20], and fibrocytes in the inner ear [16], as well as mature tissue fibroblasts including cancer-associated fibroblasts ([20, 21] and reviewed in [22, 23]), glomerular mesangial cells of the kidney [18], and fibroblasts in the valves of the heart [15].

In the present study, we sought to determine the potential of the HSC to give rise to mature lung fibroblasts and monitor the transition of these cells through the peripheral blood to their incorporation into the lung as tissue fibroblasts. Our findings demonstrated that the nonadherent fraction of bone marrow was enriched for HSC-derived cells and this population gave rise to multilineage hematopoietic engraftment. Further, we showed that the peripheral blood of wild-type mice engrafted with the nonadherent bone marrow fraction from enhanced green fluorescent protein (EGFP) mice contains EGFP^+^ circulating fibroblast precursors (CFPs). In addition, the lungs of engrafted mice were shown to contain EGFP^+^ CFPs as well as EGFP^+^ Collagen I expressing cells with a fibroblastic morphology, suggesting that nonadherent bone marrow derived circulating fibroblast precursors gave rise to mature lung fibroblasts* in vivo*. As confirmation of the HSC origin of the lung fibroblast, analysis of the lungs of clonally engrafted mice also demonstrated the presence of these cells. Thus, studies herein demonstrate that a population of lung fibroblasts is derived from the HSC in nondisease state.

## 2. Methods

### 2.1. Mice

C57Bl/6/CD45.1 breeders were from Jackson Laboratories. EGFP breeders (C57Bl/6/CD45.2 background) were provided by Dr. M. Okabe (Osaka University, Japan) [24]. Mice were bred and maintained in the Animal Research Facility, VAMC. Research was conducted in accordance with guidelines set by the US Public Health Service Policy on Humane Care and Use of Laboratory Animals and the VAMC IACUC.

### 2.2. Antibodies

Fluorochrome-conjugated, biotinylated, or purified versions of the following antibodies were used: anti-CD45R/B220 (RA3-6B2), anti-Gr-1 (anti-Ly-6G[RB6-8C5]), anti-Mac1 (anti-CD11b[M1/70]), anti-Thy-1.2 (30-H12), anti-CD45 (Leukocyte Common Antigen, Ly-5;30-F11), anti-CD45.1 (A20) from BD Biosciences; anti-DDR2 (anti-Discoidin Domain Receptor-2) (N-20) from Santa-Cruz; anti-collagen-I (ab21286), anti-CD45 (ab10558), anti-GFP (ab13970) from Abcam; isotype control antibodies from BD Biosciences/Pharmingen or Santa Cruz; all secondary antibodies from Jackson ImmunoResearch.

### 2.3. Bone Marrow Isolation and Nonadherent Bone Marrow Transplantation

Total bone marrow was harvested by flushing femurs and tibiae of C57Bl/6-EGFP/CD45.2 mice. For generation of nonadherent bone marrow, mononuclear cells were isolated using Lympholyte M (Cedar Lane) and differential centrifugation. Cells were washed in 0.1% BSA/PBS and were plated in *α*MEM containing 20% FBS and supplemented with 1% Penicillin/Streptomycin. Approximately 96 hours after isolation the nonadherent population was removed and cells were then washed and resuspended in PBS (2 × 10^6^ cells/100 *μ*L) and injected by tail vein into lethally irradiated C57Bl/6/CD45.1 recipient mice (total-body irradiation, 850 cGy). Mice were euthanized 10–12 weeks after transplant. Total EGFP^+^/CD45.1^−^ engraftment and multilineage engraftment of EGFP^+^ cells in the B cell, T cell, and granulocyte/macrophage lineages were confirmed (Table 1). For flow cytometric quantification of total engraftment, isotype controls were used to set gates for CD45.1 and EGFP populations of sized, propidium iodide negative (live) cells. In order to determine multilineage engraftment, analysis of flow cytometric data was as follows: sized, live cells were selected and gates were set on the B cell, T cell, or granulocyte/macrophage populations against isotype controls. Expression of EGFP was then analyzed from these gated populations in order to determine the percentage of EGFP expressing cells in each of the blood lineages.

### 2.4. Clonal Cell Transplantation

Clonal cell transplantation was performed as previously described [19–21, 25]. Briefly, lineage negative (Lin^−^) cells were isolated from bone marrow of 10–14-week-old C57Bl/6-EGFP/CD45.2 mice by negative selection following staining and DynaBead removal of B220, Gr-1, CD4, CD8a, and TER-119 positive cells. Lin^−^ cells were stained with antibodies to Sca-1, c-kit, and CD34. Single Lin^−^Sca-1^+^ckit^hi^CD34^−^ cells were deposited into individual wells of 96-well culture plates (MoFlo CyCLONE system). Eighteen hours after deposition, wells containing single cells were identified and cultured for 7 days in *α*-modification of Eagle's medium (*α*MEM, Life Technologies), 20% fetal bovine serum (FBS, Atlanta Biologicals), 10% bovine serum albumin (BSA, Life Technologies), 1 × 10^−2^ mol/L 2-mercaptoethanol (Sigma), 10 *μ*g/mL stem cell factor (SCF), and 10 *μ*g/mL interleukin-11 (IL-11, R&D Systems). Wells containing ≤20 clonal cells were selected for transplantation. Recipient C57Bl/6/CD45.1 mice were lethally irradiated (total-body irradiation, 950 cGy). Clonal cells were injected intravenously with 500 CD45.1/EGFP^−^/Lin^−^ckit^+^Sca-1^+^CD34^+^ radioprotective cells. Radioprotective cells are sorted EGFP negative cells that are used as short-term repopulating cells to allow the animals to survive the postirradiation pancytopenia period prior to engraftment [26]. Mice were euthanized up to one year after clonal cell transplantation. Total EGFP^+^/CD45.1^−^ engraftment and multilineage engraftment of EGFP cells in the B cell, T cell, and granulocyte/macrophage lineages was confirmed (Table 3). Flow cytometric quantification of total and multilineage engraftment was performed as described above.

### 2.5. Immunofluorescence

Lungs were harvested from engrafted animals and samples were fixed in zinc fixative (BD Biosciences) or 4% paraformaldehyde (Affymetrix). Tissues were paraffin embedded and 5 *μ*M sections cut. Citrate buffer (Vector Laboratories) mediated antigen retrieval was performed on lung tissue sections. Tissues were permeabilized in 0.02% Triton X-100/PBS, 5% serum blocked, incubated with primary antibodies listed above, and used at the following concentrations: anti-GFP 1 : 500, anti-Collagen I 1 : 50, anti-CD45 (ab10558) 1 : 100, and anti-DDR2 1 : 50. Samples were washed in PBS before incubation with fluorochrome-conjugated secondary antibodies used at 1 : 100 concentration in 5% serum. Hoechst 33342 was used as a nuclear marker. Sections were gel mounted (Electron Microscopy Sciences) and coverslipped for imaging.

### 2.6. Microscopy and Quantitative Analysis

Imaging was performed using a Nikon A1R confocal system and images were processed using NIS Elements (Nikon) and Adobe Photoshop CS5 (Adobe Systems). Fluorescent and differential interference contrast (DIC) images of lung sections from transplanted mice were taken at 400x and 600x magnification. For quantification, 2 sections with a minimum of 4 sections between were imaged at 400x for each mouse and 2 fields per section were quantified (*n* = 3 for nonadherent transplant mice and *n* = 2 for clonal cell transplant mice). Quantification of each cell population was expressed as a percent with ranges in tables. Images were shown at 600x magnification; Supplemental Figure S1 in Supplementary Material available online at http://dx.doi.org/10.1155/2015/159713 was shown at 400x magnification.

### 2.7. Flow Cytometry

Cells were resuspended in PBS and were blocked in 10% donkey serum (Jackson ImmunoResearch) for 20 minutes or were stained without block. Antibodies to cell surface proteins were added for 15 minutes at 4°C in the dark. Cells were washed and resuspended in PBS and propidium iodide was added immediately before assay on a FACs Calibur Cytometer (BD Biosciences). Isotype controls were used to set gates. Data was analyzed using FlowJo v7.6.5 and v10 (TreeStar Inc.).

### 2.8. Statistics

Analysis was conducted using Microsoft Excel. Data were presented as the mean ± standard deviation where appropriate. Student's *t*-test was used to compare groups. *P* ≤ 0.05 was regarded as statistically significant.

## 3. Results

### 3.1. Nonadherence Enriched for CD45 Expressing Cells

**Figure 1:**
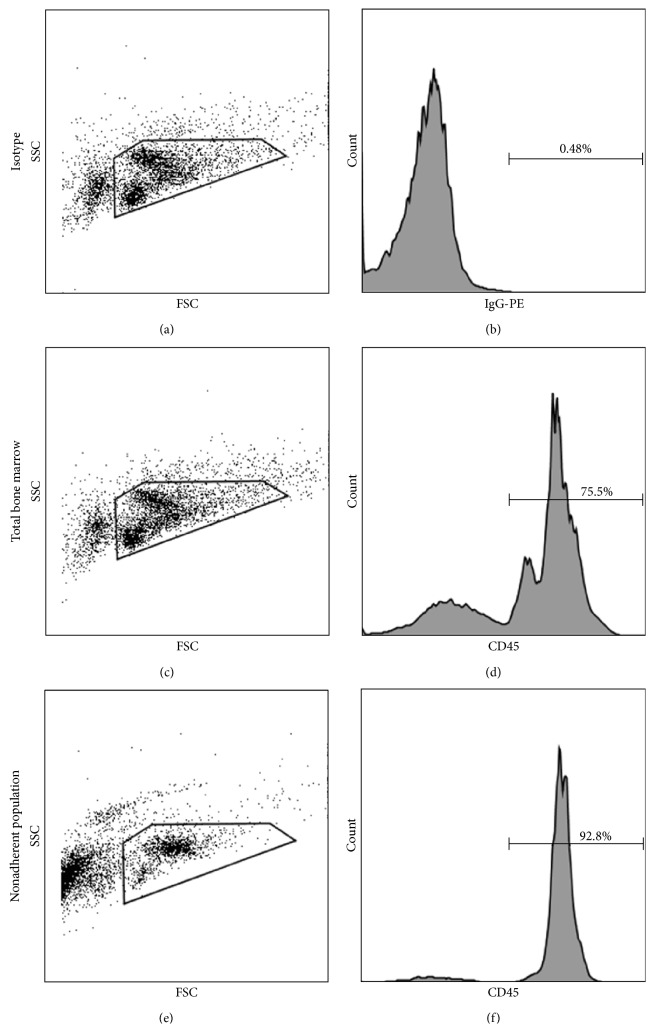
Adherence culture enriched for the HSC-derived population in the nonadherent fraction of bone marrow. Flow cytometric analysis of CD45 expression in total bone marrow was compared to that in the 96-hour nonadherent bone marrow population. A representative analysis is shown. Panel (a) shows forward and side scatter gate and Panel (b) shows isotype control for CD45 staining gated on the live, propidium iodide (PI) negative population. A representative analysis of CD45 expression by total bone marrow is shown where Panel (c) depicts the forward and side scatter gate and Panel (d) shows CD45 staining gated on the live, PI negative population. Panel (e) depicts the forward scatter and side scatter gate for the nonadherent bone marrow fraction, and Panel (f) shows the CD45 staining gated on the live, PI negative population.

Hematopoietic stem cells (HSCs) are immature cells that reside in the bone marrow and that possess the ability to give rise to colony forming cells and are capable of hematopoietic reconstitution [27–29]. These are distinct from bone marrow mesenchymal stem cells (MSCs) which are generally isolated and identified by their ability to adhere to plastic* in vitro* [30] and are characterized by their lack of expression of hematopoietic markers, including CD45 [31], the pan-leukocyte hematopoietic marker. In order to demonstrate that isolation based on nonadherence is enriched for the hematopoietic derived population, total bone marrow was first isolated from wild-type mice and analyzed for the expression of CD45 by flow cytometry. Approximately 68.4% ± SD 5.9% of the total, live, bone marrow population expressed CD45 (Figure 1). Total bone marrow was then plated on tissue culture treated flasks for 96 hours to allow for adherence. The nonadherent fraction was then removed and analyzed for expression of CD45 by flow cytometry. Quantification demonstrated that 93.4% ± SD 0.3% of the nonadherent fraction expressed CD45. This shows that the nonadherent bone marrow fraction obtained after 96 hours of adherence culture was significantly enriched for CD45 as compared to the total bone marrow population (93.4% ± SD 0.3% versus 68.4% ± SD 5.9%, resp.; ^*^ *P* = 0.002), indicating significant enrichment for the HSC-derived population (Figure 1).

### 3.2. Nonadherent Bone Marrow Transplantation Gives Rise to Multilineage Hematopoietic Engraftment

**Figure 2:**
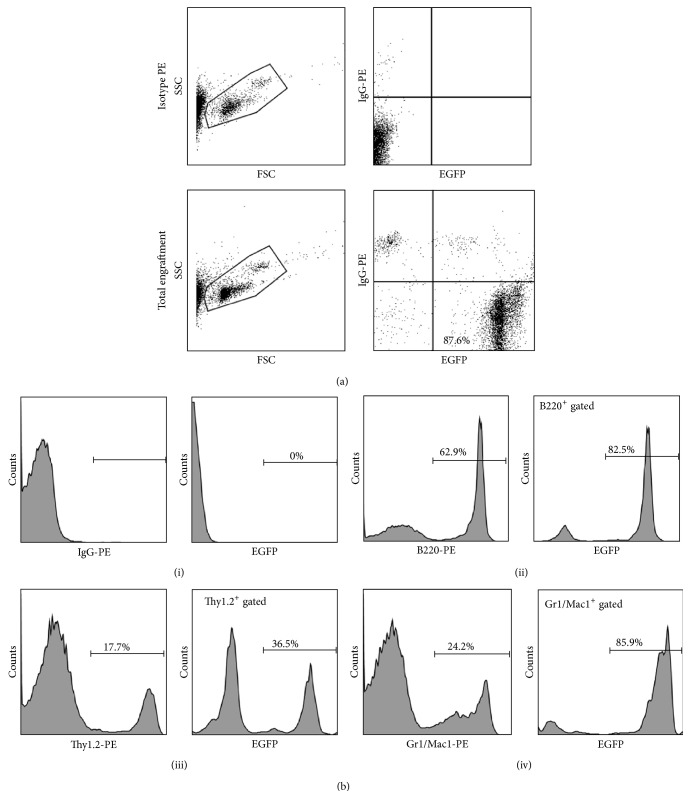
Transplantation of nonadherent bone marrow results in multilineage hematopoietic engraftment. Shown is a representative analysis of total (a) and multilineage (b) engraftment approximately 2 months after transplantation of nonadherent bone marrow cells. Isotype (top panels) forward scatter and side scatter gate is shown (left) with IgG-PE versus EGFP gate (right). Representative total engraftment is shown in Panel (a) (bottom panels) with forward scatter and side scatter gate (left) and PE and EGFP gates shown (right). (b) Representative multilineage engraftment analysis is depicted in Panel (b). The isotype IgG PE gate and the EGFP population gated on the IgG PE population are depicted in (i). Analysis of B cell (ii), T cell (iii), and granulocyte/macrophage (iv) populations demonstrates EGFP^+^ cells representing 82.5%, 36.5%, and 85.9% of each lineage, respectively. In all dot plots (i–iv), total populations are shown on the left, and EGFP^+^ cells within the gated population are shown on the right. See Table 1 for engraftment for all mice.

Mice were transplanted with bone marrow after 96 hours of selection for the nonadherent [32, 33], CD45 enriched fraction. Recipient CD45.1 mice were analyzed for total engraftment by flow cytometry 10 weeks after transplantation. High level engraftment (>50%) of CD45.1^−^/EGFP^+^ nucleated peripheral blood cells was confirmed. Total engraftment from a representative animal is depicted (Figure 2(a)). One characteristic of hematopoietic stem cells is in their capacity to repopulate all blood lineages. Therefore, as additional confirmation of enrichment of the nonadherent bone marrow fraction for HSCs [27, 29, 32–34], multilineage engraftment was confirmed in transplanted mice by flow cytometric analysis. Analysis revealed high level engraftment of EGFP^+^ cells in each of the blood lineages including the B cell (B220^+^), T cell (Thy1.2^+^), and granulocyte/macrophage (Gr1^+^/Mac1^+^) populations of nucleated peripheral blood cells at 10 weeks after transplant. Multilineage engraftment from a representative animal is shown (Figure 2(b)). Total and multilineage engraftment of each of the animals used herein is included in Table 1.

### 3.3. Circulating Fibroblast Precursors (CFPs) Arise from the Nonadherent Bone Marrow Fraction

**Figure 3:**
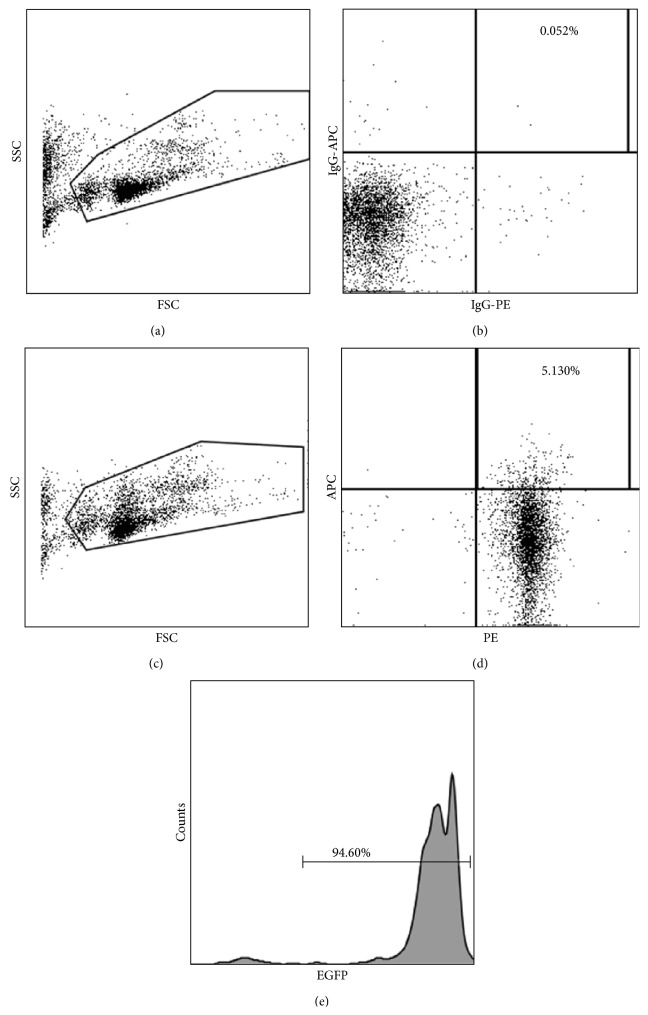
HSC-derived circulating fibroblast precursors are present in the peripheral blood of engrafted mice. Peripheral blood of engrafted nonadherent bone marrow transplanted mice was analyzed for the presence of CFPs. Representative forward scatter and side scatter gate is shown Panel (a) and isotype control is shown in Panel (b). Panels (c)–(e) depict analysis of peripheral blood from a representative animal. Forward and side scatter gate is shown in Panel (c). The population expressing CD45 (PE) and DDR2 (APC) is shown in Panel (d) (upper right quadrant). Analysis of EGFP expression of gated CD45^+^/DDR2^+^ population from Panel (d) is shown in Panel (e) (94.6%).

Our laboratory has previously identified an HSC-derived circulating fibroblast precursor (CFP) in the peripheral blood of mice transplanted with a clonal population of cells derived from a single HSC [20]. CFPs were defined by the coexpression of CD45 and Discoidin Domain Receptor-2 (DDR2) [20] and were shown to give rise to mature fibroblasts* in vitro* and tissues* in vivo* [20]. Therefore, in the present study we sought to determine whether the nonadherent bone marrow fraction could give rise to CD45^+^/DDR2^+^ CFPs in the peripheral blood* in vivo*. Flow cytometric analysis of nucleated peripheral blood cells of engrafted mice revealed 93.3% ± SD 2.5% of CD45^+^/DDR2^+^ cells coexpressed EGFP (Figure 3). Our previous studies using clonal cell transplantation indicated that ~99% of these CD45^+^/DDR2^+^ cells were derived from the HSC [20]. This data further supports the enrichment of the nonadherent bone marrow fraction for HSC-derived cells and identifies a bone marrow HSC origin of CFPs in the peripheral blood.

### 3.4. CFPs Are Present in the Lungs of Nonadherent Transplant Mice

**Figure 4:**
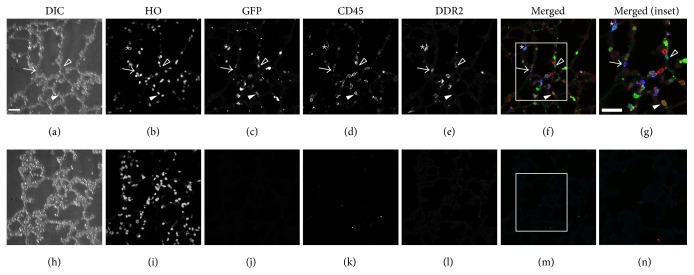
EGFP^+^ CFPs are present in the lungs of nonadherent transplant engrafted mice. Differential interference contrast (DIC), Hoechst nuclear stain (HO), GFP, CD45, and DDR2 antibody stain are shown from a representative lung section from an engrafted animal in Panels (a)–(e), respectively. Panel (f) shows merged image of GFP (green), CD45 (red), and DDR2 (blue) staining. A representative GFP^+^/CD45^+^/DDR2^+^ cell is indicated by  ∗; a representative GFP^+^/CD45^+^ cell is indicated by a solid arrow head; a representative GFP^+^/DDR2^+^ cell is indicated by an open arrow head; and a representative CD45^+^/DDR2^+^ cell is indicated by an arrow. Panel (g) shows higher magnification of inset (boxed area) in Panel (f) with ∗ indicating a GFP^+^/CD45^+^/DDR2^+^expressing cell. Panels (h)–(n) depict images of secondary only controls. Bar = 25 *μ*M.

In order to determine whether these CFPs were present in the lung, tissue sections obtained from the lungs of highly engrafted, nonadherent bone marrow transplanted mice were immunostained for fibroblast precursor markers (CD45/DDR2). Quantitative analysis of EGFP expression showed that 20.1% of cells within the lung tissue were EGFP^+^, indicating their origin from the nonadherent CD45 enriched bone marrow fraction (Figure 4, quantified in Table 2). Colocalization analysis showed expression of EGFP, CD45, and DDR2 by a subset of cells (8.59% quantitated in Table 2; representative cell indicated by ∗ in Figure 4). Cells were also identified which expressed CD45 and DDR2 (arrow) but were EGFP^−^ indicating cells that either arrived prior to bone marrow transplant or were from an alternative source.

### 3.5. Nonadherent Bone Marrow Transplant Results in Multiple EGFP^+^ Fibroblastic Populations in Lung

**Figure 5:**
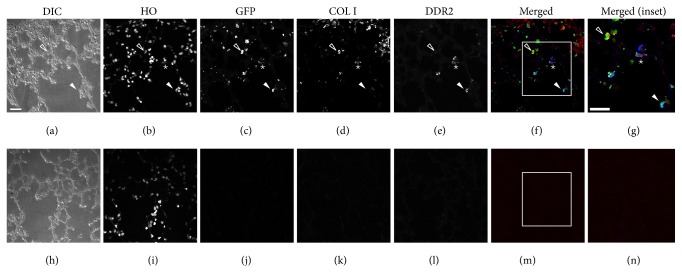
EGFP^+^ lung fibroblasts are present in the lungs of nonadherent transplant engrafted mice. Differential interference contrast (DIC), Hoechst nuclear stain (HO), GFP, Collagen I, and DDR2 antibody stain are shown from a representative lung section from an engrafted animal in Panels (a)–(e), respectively. Panel (f) shows merged image of GFP (green), Collagen I (red), and DDR2 (blue) staining. A representative GFP^+^/Collagen I^+^/DDR2^+^ cell is indicated by ∗; a representative GFP^+^/Collagen I^+^ cell is indicated by an open arrow head; a representative GFP^+^/DDR2^+^ cell is indicated by a solid arrow head. Panel (g) shows higher magnification of inset (boxed area) in Panel (f) with ∗ indicating a GFP^+^/CD45^+^/DDR2^+^expressing cell. Panels (h)–(n) depict images of secondary only controls. Bar = 25 *μ*M.

In order to determine whether nonadherent bone marrow could give rise to mature lung fibroblasts, lung tissue sections from engrafted nonadherent transplant mice were immunostained for EGFP, DDR2, and Collagen I, a known fibroblast marker in lung [1] (Figure 5 and quantified in Table 2). In the lung, 17.5% of total cells were found to express Collagen I^+^. In addition, analysis of paraffin sections revealed 3.4% of EGFP^+^ cells in the lung expressed DDR2 and Collagen I (representative cell indicated by ∗ in Figure 5). Of the EGFP^+^ cells, 16.9% coexpressed EGFP and DDR2 (representative cell indicated by the closed arrow in Figure 5, quantification in Table 2). Analysis of lung sections from nonirradiated control animals showed CD45^+^/DDR2^+^ cells with a fibroblastic morphology, indicating that the presence of HSC-derived lung fibroblasts was not an artifact of the irradiation necessary for bone marrow transplant conditioning (Supplemental Figure 1). Together, these findings demonstrate the presence of cells in the lung derived from the nonadherent fraction of bone marrow with multiple fibroblastic phenotypes.

### 3.6. HSCs Give Rise to Lung Fibroblasts

**Figure 6:**
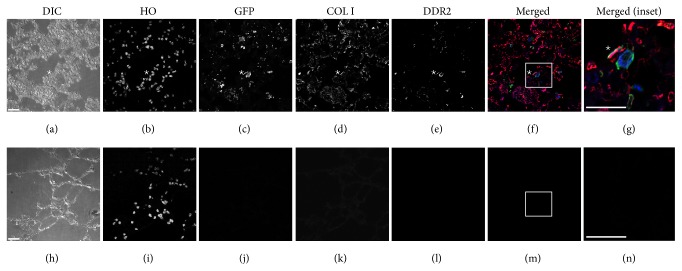
Hematopoietic stem cells give rise to lung fibroblasts* in vivo*. Differential interference contrast (DIC), Hoechst nuclear stain (HO), EGFP, Collagen I, and DDR2 antibody stain are shown in Panels (a)–(e), respectively, from a representative section of lung tissue from a clonally engrafted mouse. Panel (f) shows merged image of GFP (green), Collagen I (red), and DDR2 (blue) staining. Panel (g) shows higher magnification of inset (boxed area) in Panel (f) with ∗ indicating a GFP/Collagen I/DDR2 expressing cell. Panels (h)–(n) depict images from secondary only staining controls.

As confirmation of the specific HSC origin of lung fibroblasts, mice engrafted with a clonal population derived from a single sorted HSC were generated as described previously [19–21, 25]. Total CD45.1^−^/EGFP^+^ engraftment and multilineage engraftment were confirmed (Table 3). Lungs were harvested and paraffin embedded and sections were stained for DDR2 and Collagen I to identify fibroblasts derived from the HSC. Numerous EGFP^+^ cells expressing DDR2 (28.8%) or Collagen I (13.5%) were identified (Figure 6 and quantified in Table 4), as were EGFP^+^ cells that expressed Collagen I and DDR2 (5.6%, representative cell indicated by ∗ in Figure 6) confirming an HSC origin of a population of lung fibroblasts.

## 4. Discussion

Bone marrow stem cell populations have been shown to give rise to fibroblasts in multiple tissues in normal and disease states and our previous studies demonstrate an HSC origin for fibroblasts [15, 16, 18, 21, 35] and their precursors [19, 20]. To our knowledge, no direct lineage tracing studies have previously been performed examining the origin of lung fibroblasts* in vivo*. In the present study, we add to our previous findings of HSC plasticity by demonstrating that HSCs give rise to a functional lung fibroblast population and show their transition through a circulating fibroblast precursor to a mature lung fibroblast. It should be noted that, due to the turnover rate of lung fibroblasts in nondisease state [36], the fraction of HSC-derived fibroblastic cells observed herein in transplanted animals is likely an underrepresentation of the total HSC-derived lung fibroblast population. The presence of EGFP^+^/CD45^+^/DDR2^+^ cells in both the blood and lung tissue of engrafted mice suggests that CFPs leave the circulation, enter the lung (with a newly arriving cell retaining the CFP phenotype [20]), and incorporate into the lung tissue. Immunofluorescent characterization of EGFP^+^ HSC-derived cells in the lung tissues revealed the presence of cells with multiple phenotypes. The observation of HSC-derived CD45^+^/DDR2^+^ cells (CFPs) as well as HSC-derived CD45^−^ cells that expressed DDR2^+^ and/or Collagen I^+^ cells suggests a cell in transition. This phenotype is in line with our previous studies that have demonstrated the* in vitro* transition of bone marrow from an immature phenotype to a fibroblastic phenotype with gradual loss of CD45 expression and gain of DDR2 expression and Collagen I expression [19]. Interestingly, we also observed multiple phenotypes with respect to DDR2 and Collagen I expression in lung fibroblasts, including EGFP^+^/DDR2^−^/Collagen I^+^, EGFP^+^/DDR2^+^/Collagen I^−^, and EGFP^+^/DDR2^+^/Collagen I^+^, suggesting heterogeneity of the HSC-derived lung fibroblast population. Given that DDR2 can serve as a matrix sensing Collagen receptor [37–39], it is possible that the heterogeneous expression of Collagen I and DDR2 is reflective of the function of these cells in steady state.

While the mechanisms regulating the participation, incorporation, and phenotype of HSC-derived cells in the lung remain to be determined, our data suggest that the HSC is a significant and continual contributor to the lung fibroblast population and may have a potential role in disease. Our previous studies in solid tumor have shown that a single HSC can give rise to a significant portion of stromal fibroblasts in disease state [20, 21], suggesting that the HSC-derived population of lung fibroblasts may indeed increase with disease ([36] and reviewed in [40]). The plasticity of the HSC and presence of circulating intermediates suggest that both the HSC and CFP may be novel and exciting targets for the therapeutic treatment of lung pathologies. Particularly relevant here, the circulating fibroblast precursor population introduces the potential to target this novel HSC-derived precursor prior to its incorporation as mature lung fibroblasts. While fibrocytes have been implicated to have a role in lung diseases, they represent only a small portion of circulating leukocytes (0.1–0.5%) [41]. The relative size of this population brings into question the potential for this population to be the sole source of recruited fibroblast precursors/fibroblasts in disease such as pulmonary fibrosis. Our data suggests that targeting the CFP may affect a larger proportion of cells capable of giving rise to lung fibroblasts. Further studies of the role of HSC-derived cells* in vivo*, factors regulating their recruitment and fate, and the ability to be targeted in disease models, are necessary to determine the full contribution of the HSC to lung pathophysiology.

## Supplementary Material

HSC-derived lung fibroblasts are not an artifact of irradiation. Differential interference contrast (DIC), Hoechst nuclear stain (HO), CD45, DDR2 antibody stain are shown in Panels A–D, respectively, from a representative section of lung tissue from a non-irradiated mouse. Panel E shows merged image of HO (blue), CD45 (green), DDR2 (red) staining. Panel F shows higher magnification of inset (boxed area) in Panel E with ^*^ indicating a CD45/DDR2 expressing cell (green and red). Panels G–L depict images from secondary only staining controls. 400x magnification, mag bar = 25 *μ*M

## Figures and Tables

**Table 1:** Nonadherent fraction bone marrow transplantation results in multilineage hematopoietic engraftment.

	Total engraftment (%CD45.1^−^/EGFP^+^)	B cell engraftment (%B220^+^/EGFP^+^)	T cell engraftment (%Thy1.2^+^/EGFP^+^)	Granulocyte/macrophage engraftment (%Gr1/Mac1^+^/EGFP^+^)
M1	80.8	81.7	36.1	81.4
M2	87.6	82.5	36.5	85.9
M3	85.7	90.6	44.4	84.6
M4	55.2	60.0	22.9	60.0
M5	84.4	80.5	41.4	81.5

M1–M5 represent five individual mice transplanted with EGFP^+^ nonadherent bone marrow. As we have previously noted, the low level T cell engraftment reflects slow turnover of T cells *in vivo*.

**Table 2:** Heterogeneity of EGFP cells in lungs of transplanted animals.

	EGFP	EGFP CD45/DDR2	EGFP Col I/DDR2	EGFP Col I	EGFP DDR2
Nonadherent transplant	25.0%	8.6%	3.4%	14.1%	16.9%
	(16.9%–31.3%)	(2.4%–18.0%)	(0%–6.4%)	(4.2%–40.0%)	(6.4%–26.8%)

Average percentage in each phenotype is indicated in top row. Bottom row gives range of percentages of each phenotype.

**Table 3:** Total and multilineage hematopoietic engraftment from a clonal population derived from a single EGFP^+^ HSC.

	Total engraftment (%CD45.1^−^/EGFP^+^)	B cell engraftment (%B220^+^/EGFP^+^)	T cell engraftment (%Thy1.2^+^/EGFP^+^)	Granulocyte/macrophage engraftment (%Gr1/Mac1^+^/EGFP^+^)
M1	55.1	76.2	28.3	87.9
M2	93.6	98.5	62.4	99.8

M1 and M2 represent two animals transplanted with a clonal population derived from a single sorted EGFP^+^ HSC.

**Table 4:** HSCs give rise to lung fibroblasts.

	EGFP	EGFP Col I/DDR2	EGFP Col I	EGFP DDR2
Clonal cell transplant	21.1%	5.6%	13.5%	28.8%
	(7.9%–44.0%)	(0.1%–8.7%)	(9.1%–23.1%)	(20.0%–42.3%)

Average percentage in each phenotype is indicated in top row; bottom row gives range of percentages of each phenotype.

## References

[1] Hashimoto N., Jin H., Liu T., Chensue S. W., Phan S. H. (2004). Bone marrow-derived progenitor cells in pulmonary fibrosis. *The Journal of Clinical Investigation*.

[2] Epperly M. W., Guo H., Gretton J. E., Greenberger J. S. (2003). Bone marrow origin of myofibroblasts in irradiation pulmonary fibrosis. *The American Journal of Respiratory Cell and Molecular Biology*.

[3] Bucala R., Spiegel L. A., Chesney J., Hogan M., Cerami A. (1994). Circulating fibrocytes define a new leukocyte subpopulation that mediates tissue repair. *Molecular Medicine*.

[4] Schmidt M., Sun G., Stacey M. A., Mori L., Mattoli S. (2003). Identification of circulating fibrocytes as precursors of bronchial myofibroblasts in asthma. *The Journal of Immunology*.

[5] Lama V. N., Phan S. H. (2006). The extrapulmonary origin of fibroblasts: stem/progenitor cells and beyond. *Proceedings of the American Thoracic Society*.

[6] Xia H., Bodempudi V., Benyumov A. (2014). Identification of a cell-of-origin for fibroblasts comprising the fibrotic reticulum in idiopathic pulmonary fibrosis. *American Journal of Pathology*.

[7] Willis B. C., DuBois R. M., Borok Z. (2006). Epithelial origin of myofibroblasts during fibrosis in the lung. *Proceedings of the American Thoracic Society*.

[8] Hashimoto N., Phan S. H., Imaizumi K. (2010). Endothelial-mesenchymal transition in bleomycin-induced pulmonary fibrosis. *American Journal of Respiratory Cell and Molecular Biology*.

[9] Hung C., Linn G., Chow Y.-H. (2013). Role of lung pericytes and resident fibroblasts in the pathogenesis of pulmonary fibrosis. *American Journal of Respiratory and Critical Care Medicine*.

[10] Hutchison N., Fligny C., Duffield J. S. (2013). Resident mesenchymal cells and fibrosis. *Biochimica et Biophysica Acta*.

[11] Ogawa M., Larue A. C., Watson P. M., Watson D. K. (2010). Hematopoietic stem cell origin of mesenchymal cells: opportunity for novel therapeutic approaches. *International Journal of Hematology*.

[12] Ogawa M., LaRue A. C., Watson P. M., Watson D. K. (2010). Hematopoietic stem cell origin of connective tissues. *Experimental Hematology*.

[13] Catacchio I., Berardi S., Reale A. (2013). Evidence for bone marrow adult stem cell plasticity: properties, molecular mechanisms, negative aspects, and clinical applications of hematopoietic and mesenchymal stem cells transdifferentiation. *Stem Cells International*.

[14] Shirai K., Sera Y., Bulkeley W. (2009). Hematopoietic stem cell origin of human fibroblasts: cell culture studies of female recipients of gender-mismatched stem cell transplantation and patients with chronic myelogenous leukemia. *Experimental Hematology*.

[15] Visconti R. P., Ebihara Y., LaRue A. C. (2006). An in vivo analysis of hematopoietic stem cell potential: hematopoietic origin of cardiac valve interstitial cells. *Circulation Research*.

[16] Lang H., Ebihara Y., Schmiedt R. A. (2006). Contribution of bone marrow hematopoietic stem cells to adult mouse inner ear: mesenchymal cells and fibrocytes. *Journal of Comparative Neurology*.

[17] Ishikawa F., Drake C. J., Yang S. (2003). Transplanted human cord blood cells give rise to hepatocytes in engrafted mice. *Annals of the New York Academy of Sciences*.

[18] Masuya M., Drake C. J., Fleming P. A. (2003). Hematopoietic origin of glomerular mesangial cells. *Blood*.

[19] Ebihara Y., Masuya M., Larue A. C. (2006). Hematopoietic origins of fibroblasts: II. In vitro studies of fibroblasts, CFU-F, and fibrocytes. *Experimental Hematology*.

[20] Abangan R. S., Williams C. R., Mehrotra M., Duncan J. D., LaRue A. C. (2010). MCP1 directs trafficking of hematopoietic stem cell-derived fibroblast precursors in solid tumor. *The American Journal of Pathology*.

[21] LaRue A. C., Masuya M., Ebihara Y. (2006). Hematopoietic origins of fibroblasts: I. In vivo studies of fibroblasts associated with solid tumors. *Experimental Hematology*.

[22] Ogawa M., LaRue A. C., Drake C. J. (2006). Hematopoietic origin of fibroblasts/myofibroblasts: its pathophysiologic implications. *Blood*.

[23] McDonald L. T., LaRue A. C. (2012). Hematopoietic stem cell derived carcinoma-associated fibroblasts: a novel origin. *International Journal of Clinical and Experimental Pathology*.

[24] Nakanishi T., Kuroiwa A., Yamada S. (2002). Fish analysis of 142 EGFP transgene integration sites into the mouse genome. *Genomics*.

[25] Mehrotra M., Williams C. R., Ogawa M., LaRue A. C. (2013). Hematopoietic stem cells give rise to osteo-chondrogenic cells. *Blood Cells, Molecules, and Diseases*.

[26] Osawa M., Hanada K.-I., Hamada H., Nakauchi H. (1996). Long-term lymphohematopoietic reconstitution by a single CD34-low/negative hematopoietic stem cell. *Science*.

[27] Fricke S., Fricke C., Oelkrug C. (2010). Characterization of murine non-adherent bone marrow cells leading to recovery of endogenous hematopoiesis. *Cellular and Molecular Life Sciences*.

[28] Lebkowski J. S., McNally M. A., Finch S. (1990). Enrichment of murine hematopoietic stem cells. Reconstitution of syngeneic and haplotype-mismatched mice. *Transplantation*.

[29] Kiefer F., Wagner E. F., Keller G. (1991). Fractionation of mouse bone marrow by adherence separates primitive hematopoietic stem cells from in vitro colony-forming cells and spleen colony-forming cells. *Blood*.

[30] Friedenstein A. J., Deriglasova U. F., Kulagina N. N. (1974). Precursors for fibroblasts in different populations of hematopoietic cells as detected by the in vitro colony assay method. *Experimental Hematology*.

[31] Pittenger M. F., Mackay A. M., Beck S. C. (1999). Multilineage potential of adult human mesenchymal stem cells. *Science*.

[32] Zhang Z. L., Tong J., Lu R. N., Scutt A. M., Goltzman D., Miao D. S. (2009). Therapeutic potential of non-adherent BM-derived mesenchymal stem cells in tissue regeneration. *Bone Marrow Transplantation*.

[33] Otsuru S., Gordon P. L., Shimono K. (2012). Transplanted bone marrow mononuclear cells and MSCs impart clinical benefit to children with osteogenesis imperfecta through different mechanisms. *Blood*.

[34] Fricke S., Ackermann M., Stolzing A. (2009). Allogeneic non-adherent bone marrow cells facilitate hematopoietic recovery but do not lead to allogeneic engraftment. *PLoS ONE*.

[35] Hess D. C., Abe T., Hill W. D. (2004). Hematopoietic origin of microglial and perivascular cells in brain. *Experimental Neurology*.

[36] Clark J. G., Kostal K. M., Marino B. A. (1982). Modulation of collagen production following bleomycin-induced pulmonary fibrosis in hamsters. Presence of a factor in lung that increases fibroblast prostaglandin E2 and cAMP and suppresses fibroblast proliferation and collagen production. *The Journal of Biological Chemistry*.

[37] Ruiz P. A., Jarai G. (2012). Discoidin domain receptors regulate the migration of primary human lung fibroblasts through collagen matrices. *Fibrogenesis and Tissue Repair*.

[38] Kim D., You E., Min N. Y., Lee K.-H., Kim H. K., Rhee S. (2013). Discoidin domain receptor 2 regulates the adhesion of fibroblasts to 3D collagen matrices. *International Journal of Molecular Medicine*.

[39] Olaso E., Labrador J.-P., Wang L. (2002). Discoidin domain receptor 2 regulates fibroblast proliferation and migration through the extracellular matrix in association with transcriptional activation of matrix metalloproteinase-2. *The Journal of Biological Chemistry*.

[40] Clarke D. L., Carruthers A. M., Mustelin T., Murray L. A. (2013). Matrix regulation of idiopathic pulmonary fibrosis: the role of enzymes. *Fibrogenesis and Tissue Repair*.

[41] Abe R., Donnelly S. C., Peng T., Bucala R., Metz C. N. (2001). Peripheral blood fibrocytes: differentiation pathway and migration to wound sites. *The Journal of Immunology*.

