# Supplementary material for: Hematopoietic Origin of Murine Lung Fibroblasts
Source: Stem Cells Int. 2015 Jun 21;2015:159713. doi: 10.1155/2015/159713 (PMC4491389; doi:10.1155/2015/159713)
Supplement: Supplementary file 1 — HSC-derived lung fibroblasts are not an artifact of irradiation. Differential interference contrast (DIC), Hoechst nuclear stain (HO), CD45, DDR2 antibody stain are shown in Panels A–D, respectively, from a representative section of lung tissue from a non-irradiated mouse. Panel E shows merged image of HO (blue), CD45 (green), DDR2 (red) staining. Panel F shows higher magnification of inset (boxed area) in Panel E with * indicating a CD45/DDR2 expressing cell (green and red). Panels G–L depict images from secondary only staining controls. 400x magnification, mag bar = 25 μM [file 159713.f1.pdf]

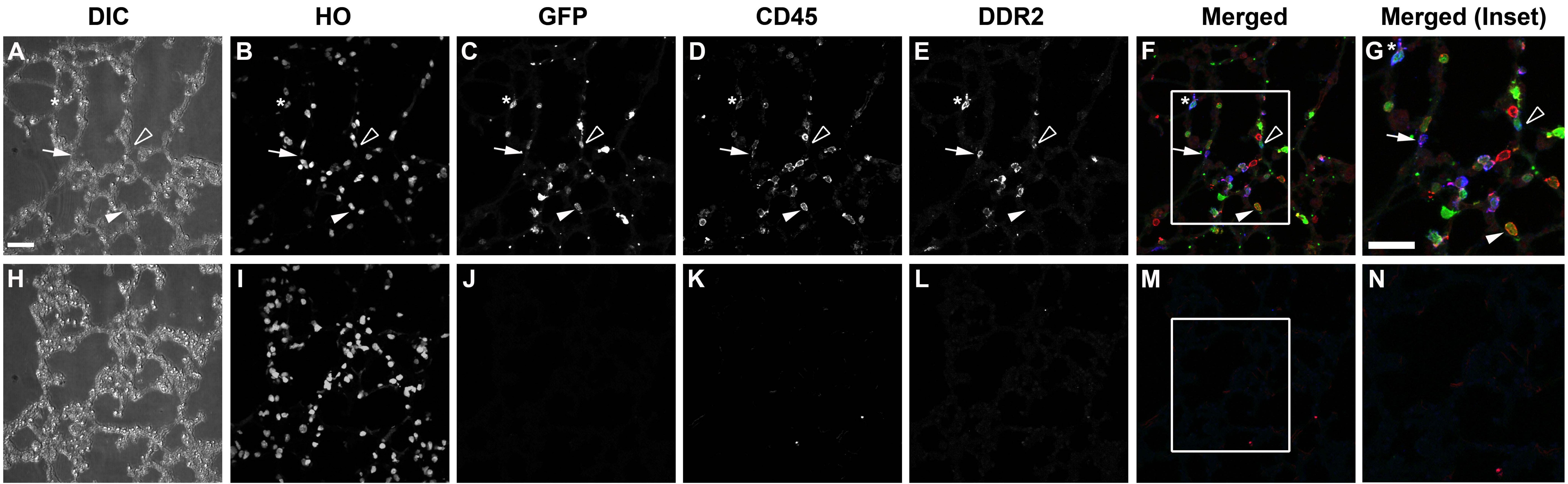
DIC
HO
GFP
CD45
DDR2
Merged
Merged (Inset)
A
B
C
D
E
F
G
H
I
J
K
L
M
N

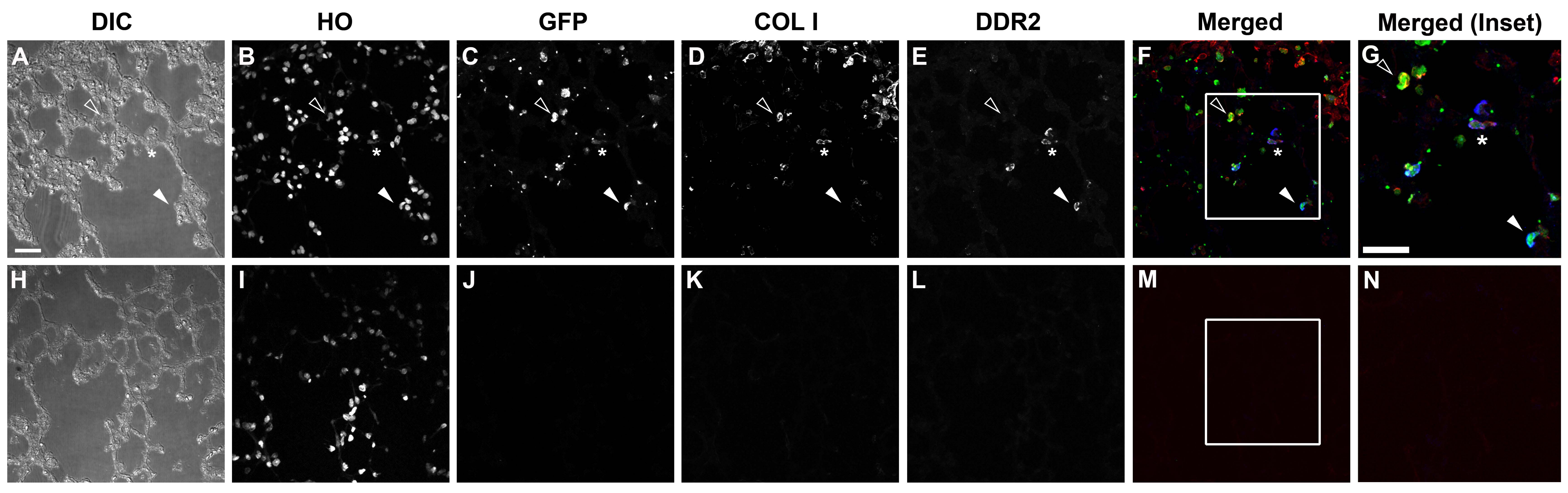
DIC
HO
GFP
COL I
DDR2
Merged
Merged (Inset)
A
B
C
D
E
F
G
H
I
J
K
L
M
N

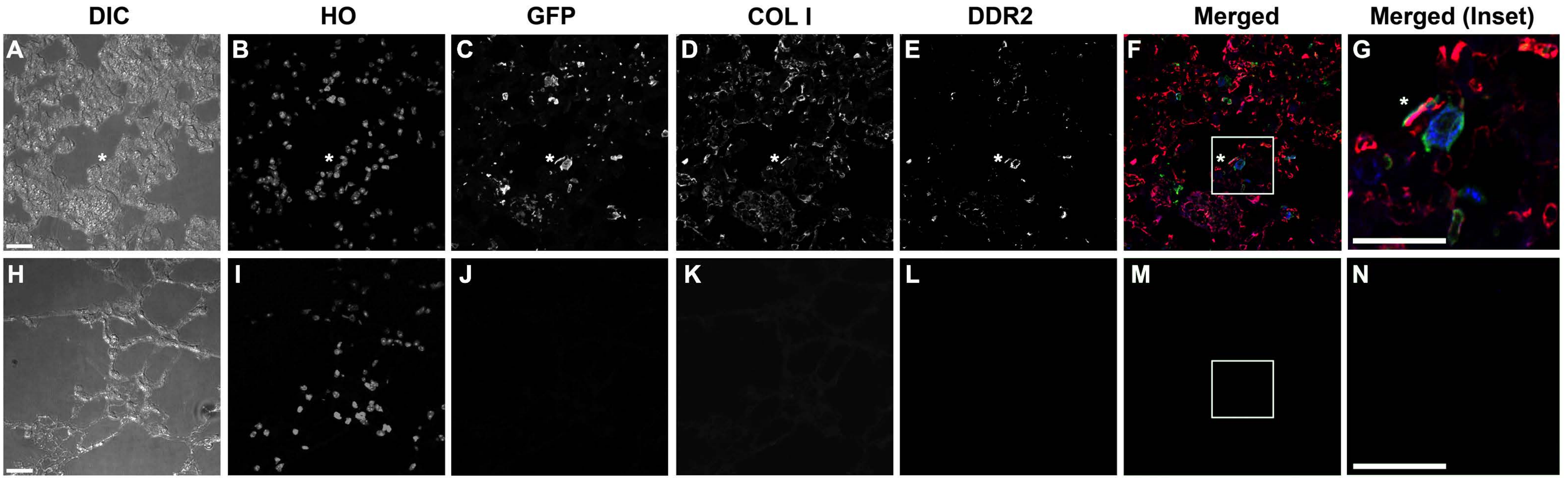
DIC
HO
GFP
COL I
DDR2
Merged
Merged (Inset)
A
B
C
D
E
F
G
H
I
J
K
L
M
N

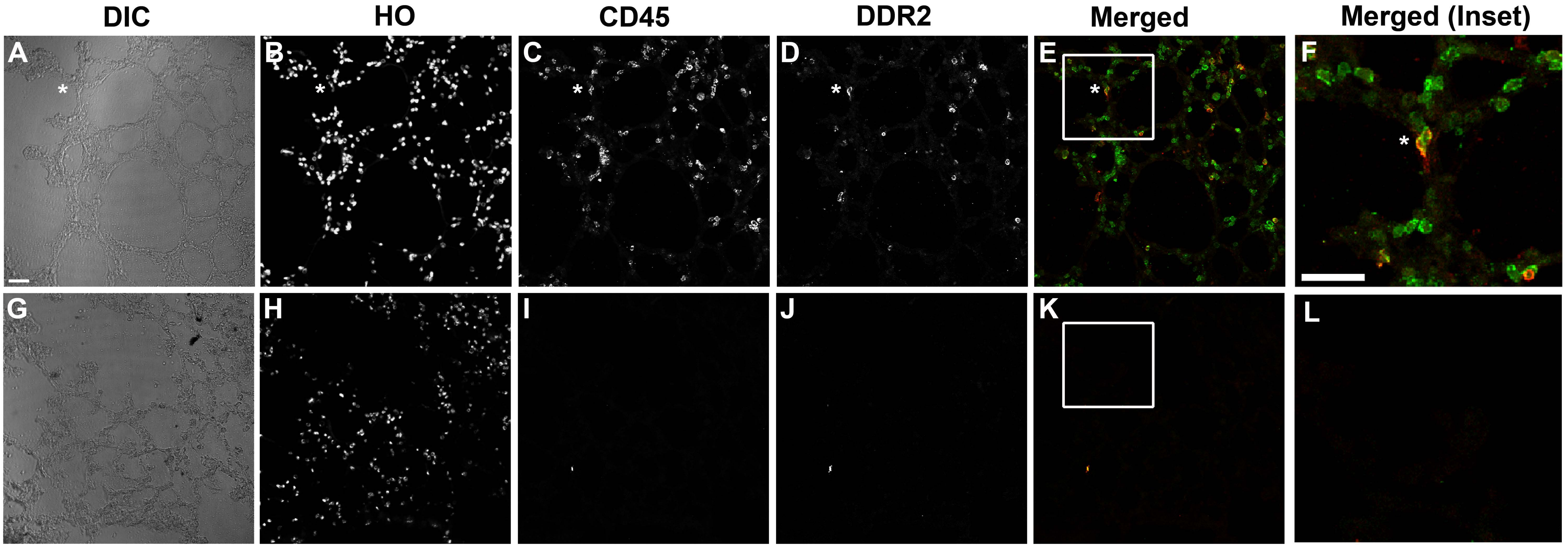

DIC
HO
CD45
DDR2
Merged
Merged (Inset)
A
B
C
D
E
F
G
H
I
J
K
L
